# Supplementary material for: Molecular motion of a nanoscopic moonlander via translations and rotations of triphenylphosphine on graphite
Source: Commun Chem. 2024 Apr 6;7:78. doi: 10.1038/s42004-024-01158-7 (PMC10998885; doi:10.1038/s42004-024-01158-7)
Supplement: Supplementary file 2 — Supplementary Information [file 42004_2024_1158_MOESM2_ESM.pdf]

Supplementary information: Molecular motion of a nanoscopic moonlander via translations and rotations of triphenylphosphine on graphite.

Anton Tamtögl,*,[1] Marco Sacchi,[2] Victoria Schwab,[1] Michael M. Koza,[3] and Peter Fouquet[3]

[1] *Institute of Experimental Physics, Graz University of Technology, Graz, Austria*
[2] *Department of Chemistry, University of Surrey, GU2 7XH, Guildford, United Kingdom*
[3] *Institut Laue-Langevin, 71 Avenue des Martyrs, 38000 Grenoble, France*
* E-mail: tamtoegl@tugraz.at

Supplementary methods

Further details on the fitting and analysis are given in the following. Supplementary Figure 1 shows a contour plot of the dynamic scattering functions $S(Q, \Delta E)$ in (a) as well as several $S(Q = const, \Delta E)$ in (b) as extracted from the neutron TOF data for exfoliated graphite covered by 0.5 ML of PPh_3. The solid lines show the quasi-elastic Lorentzian broadenings based on fitting Equation 1 of the main manuscript.

Supplementary Figure 2 shows two NSE measurements. As shown in the upper panel, almost all temperatures and coverages are best described by a single exponential decay that is used as a model function for the data fitting. We note however, that at a sample temperature of 350 K the low Q data at 0.9 ML coverage is better described by a fit with $\beta < 1$ (lower panel of Supplementary Figure 2). The effect becomes less pronounced at 420 K, whereas at 500 K it tends to be well described with a single exponential fit again. Such behaviour is common if two different dynamical processes are present and it becomes particularly evident at the temperature where the transition from intramolecular motion to the translational motion occurs,[1] as described in the main part of the manuscript.

The low-temperature TOF data for 0.5 ML is further shown in Supplementary Figure 3(a), illustrating that some dynamics is already present at low temperature, although the uncertainties are too large to draw any reasonable conclusions. As mentioned in the main text, the quality of the neutron TOF data gives rise to large uncertainties of the extracted broadenings and for $T < 270$ K the data is accordingly scattered over the Q-range. Moreover, the Q-dependence of the elastic NSE amplitude y_0 as shown in Supplementary Figure 3(b) for 0.9 ML clearly indicates that the low temperature motion is associated with an rotational / flapping motion of the phenyl groups.

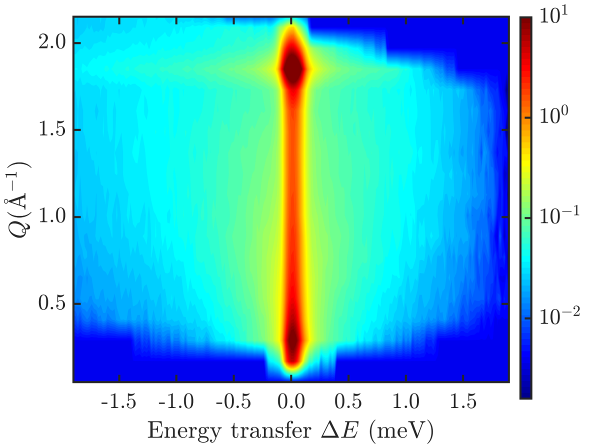

(a) Two-dimensional contour plot of the dynamic scattering function $S(Q, \Delta E)$ that was extracted from neutron TOF data obtained for exfoliated graphite covered by 0.5 ML of $P(C_6H_5)_3$ at 500 K. The intense spot at about $Q = 1.9$ Å^{-1} is due to the (002) Bragg reflection from the basal plane of graphite.

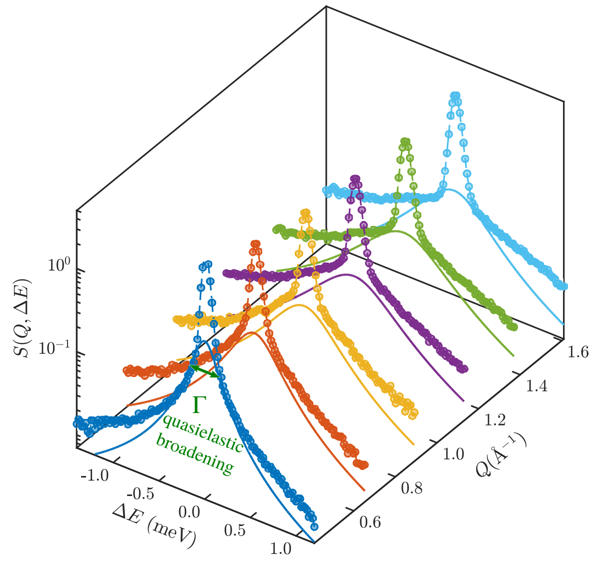

(b) Several dynamic scattering functions $S(Q, \hbar\omega)$ at constant Q as extracted from neutron TOF data plotted as symbols. The solid lines show the fitted quasi-elastic broadening (Γ, note the logarithmic scale) obtained by fitting a Lorentzian peak convoluted with the experimental resolution according to Equation 1 of the main manuscript.

Supplementary Figure 1: Neutron TOF spectra for 0.5 ML of $P(C_6H_5)_3$ adsorbed on graphite, after conversion to $S(Q, \Delta E)$.

Supplementary DFT calculations

In Supplementary Table 1 we present the full set of DFT calculations, i.e. the data for all adsorption geometries / configurations of PPh_3 on graphite which have been calculated using van-der-Waals corrected DFT. The nomenclature of the adsorption site / position and the rotation of the molecule follow the same principle as described in the main part of the manuscript.

Supplementary Table 1: The adsorption energy E_a and the energy difference ΔE_a relative to the most favourable adsorption site for PPh_3 adsorption on graphite with the phenyl groups pointing towards the surface (Configuration down) and pointing away from the surface (Configuration up), respectively. The 9 different adsorption sites within the graphite unit cell (position 1-9 in Figure 1(b) of the main text) are referred to the C_3 rotational axis through the P atom. The last column shows the energy difference ΔE_{inv} for inversion of the molecule from the downwards configuration to the upwards configuration.

		Configuration down		Configuration up		Inversion
Position	Rotation (°)	E_a (eV)	ΔE_a (meV)	E_a (eV)	ΔE_a (meV)	ΔE_{inv} (meV)
1	0	−3.042	70	−2.925	73	117
2	0	−3.075	37	−2.927	71	148
3	0	−3.099	13	−2.913	85	186
4	0	−3.081	31	−2.958	40	123
5	0	−3.102	10	−2.953	46	150
6	0	−3.081	32	−2.932	66	149
7	0	−3.100	12	−2.994	4	106
8	0	−3.073	39	−2.951	48	123
9	0	−3.038	74	−2.925	73	113
1	30	−3.076	36	−2.905	93	171
2	30	−3.072	40	−2.935	63	137
3	30	−3.059	53	−2.957	41	102
4	30	−3.086	26	−2.940	58	146
5	30	−3.081	32	−2.960	38	121
6	30	−3.081	32	−2.932	66	148
7	30	−3.099	13	−2.963	35	136
8	30	−3.082	30	−2.940	58	142
9	30	−3.081	32	−2.901	97	179
1	60	−3.111	1	−2.912	87	200
2	60	−3.078	34	−2.926	72	152
3	60	−3.043	69	−2.936	62	107
4	60	−3.092	20	−2.953	45	139
5	60	−3.066	46	−2.952	46	114
6	60	−3.084	28	−2.930	68	154
7	60	−3.104	9	−2.998	0	105
8	60	−3.100	13	−2.947	51	153
9	60	−3.112	0	−2.908	90	204

Supplementary coverage dependence analysis

An analysis of the 0.2 ML high-temperature TOF data in analogy to the combined 0.5 ML TOF and NSE data is shown below. Supplementary Figure 4 shows the extracted broadening $\Gamma(Q)$ for 0.2 ML of PPh_3 which is fitted to Equation 4 of the main text (dashed lines). The extract diffusion parameters for mass transport of 0.2 ML PPh_3 on graphite are summarised in Supplementary Table 2. While the uncertainties are larger compared to the 0.5 ML data, as we do not have NSE data for the low-Q region at the current coverage, it appears to indicate that D becomes slightly larger compared to the 0.5 ML data. As already mentioned in the main text we anticipate that inter-molecular interactions and simple site blocking slow the diffusion down with increasing coverage to a small extend.

At the same time the constant offset upon $Q \to 0$ seems to narrowly increase with increasing coverage which may indicate that the confined motion, i.e. the rotation and flapping motion of the phenyl groups becomes also again more important with coverage. The latter is in line with the Q-dependence of the elastic NSE amplitude y_0 as shown in Supplementary Figure 3(b) for 0.9 ML. Compared to the 0.5 ML data in Figure 3(a) it appears that the contribution from rotational motion increases with coverage. Consequently, as one may anticipate, with further increasing coverage, translation becomes slightly hindered, while at the same time confined i.e. local motion of the individual molecules including the mentioned rotation and flapping of the phenyl groups becomes more important also at higher temperatures.

Supplementary Table 2: Diffusion parameters according to fitting Equation 5 in the main text to the 0.2 ML neutron TOF data as shown in Supplementary Figure 4 in the $350 - 500$ K temperature range.

T (K)	l (Å)	τ (ps)	D (m^2s^{-1})
345	3.5 ± 0.1	4.3 ± 0.3	$(12 \pm 2) \cdot 10^{-10}$
413	3.3 ± 0.1	2.8 ± 0.1	$(16 \pm 1) \cdot 10^{-10}$
495	3.25 ± 0.04	2.4 ± 0.04	$(18 \pm 1) \cdot 10^{-10}$

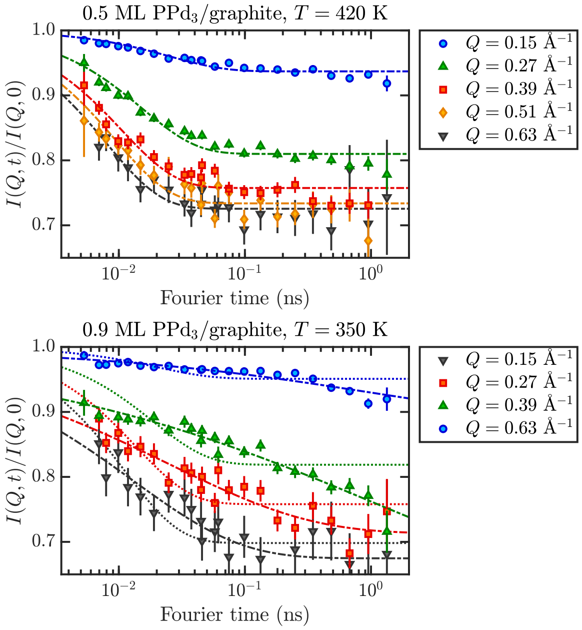

Supplementary Figure 2: $I(Q,t)/I(Q,0)$ for both coverages. Upper panel: 0.5 ML data, fitted with a single exponential fit (Equation 2 of the main text with $\beta = 1$, dash-dotted lines). Lower panel: The 0.9 ML data around 350 K is better described by Equation 2 with $\beta < 1$ (dash-dotted lines) in contrast to a single exponential fit (dashed lines).

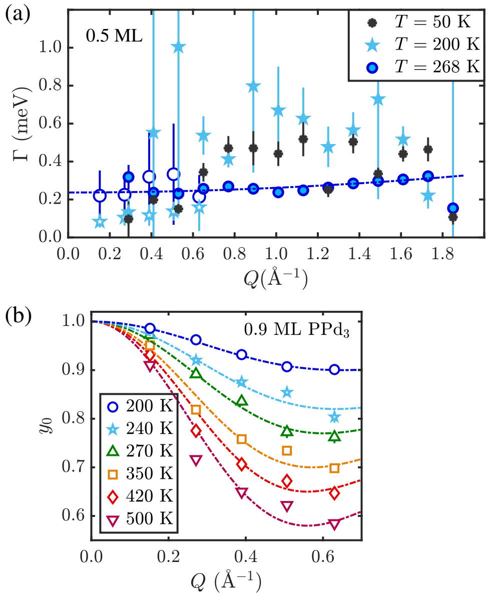

Supplementary Figure 3: (a) Full Q-dependence of the broadenings Γ in the low-temperature regime, as extracted from the neutron TOF data of the 0.5 ML sample. The uncertainty is relatively large up to about 270 K, and consequently the data is quite scattered over the Q-range. As illustrated in the main manuscript, the quasi-elastic intensity increases nevertheless continuously with temperature. (b) NSE data taken at 0.9 ML in analogy to the 0.5 ML data of Figure 3(a) in the main text. The decrease of the elastic contribution y_0 with Q is again well described by Equation 4 with a radius that equals roughly the size of a phenyl group. We further note that at 0.9 ML the data seems to be well fitted up to about 420 K and thus with increasing coverage the rotational contribution seems to increase also at higher temperatures (see text).

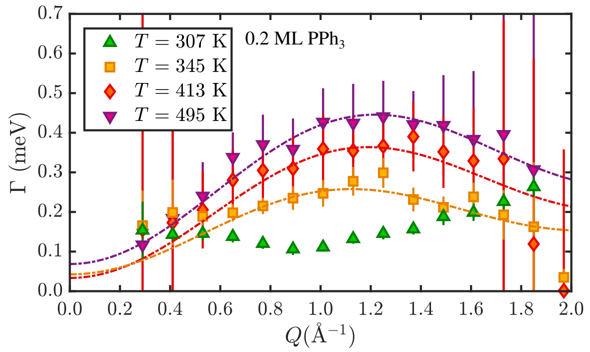

Supplementary Figure 4: The extracted quasi-elastic broadenings $\Gamma(Q)$ for 0.2 ML PPh_3 in the high-temperature regime versus momentum transfer Q. Extraction of the corresponding diffusion parameters follows from fitting Equation 5 as described in the main text. However, because there is no additional NSE data in the small Q-range available for the same coverage, the confidence bounds obtained by the fit are larger compared to the 0.5 ML data.

Supplementary details of the force field MD simulations

The simulation was performed in the Software Materials Studio[2] using the Forcite module. A graphite lattice of 4 layers was built in a 3D atomistic environment and the PPh_3 molecules were set manually above the surface at a distance of 9 Å. As the PPh_3 molecules possess a polar surface area of about 63 Å^2, 13 molecules were set on the surface with an area of $\approx$ 1698 Å^2 in order to simulate a 0.5 monolayer coverage system.

The specific 0.5 ML coverage system was chosen for the simulation since most of the experimental data in the TOF and SE measurements was acquired for the same coverage. For the graphite layers, a triclinic unit cell was built and the whole system was placed in a vacuum, using periodic boundary conditions for the entire system (Supplementary Figure 5). The first step of the simulation consisted of performing a geometry optimisation in order to guarantee that the simulation does not start from a high-energy state. This was done by applying the Smart algorithm which uses a cascade of the steepest descent, ABNR, and quasi-Newton methods. A snapshot of the setup for the MD calculations is shown in Supplementary Figure 5.

In the following, the molecular dynamics simulation was performed in Materials Studio at different temperatures. Simulations were run with the canonical or NVT ensemble which keeps the volume and the number of particles constant and allows to control the temperature by using a thermostat. The Nosé-Hoover-Langevin (NHL) thermostat was chosen because it leads to rapid convergence in the simulation. The NHL-thermostat is a refinement of the Nosé-Hoover thermostat that adds a fictitious dynamic parameter to the equations of motion in order to let the system equilibrate at the desired temperature and simulates the coupling of the system to a heat bath. Hence, comparison of the simulation to the experimental results is easily obtained in the NVT ensemble with the NHL thermostat, keeping the system at the desired temperature.

The recently developed COMPASS III force field[3] was applied and the atom charges were set to be assigned by the force field. For the electrostatic interactions, the Ewald summation method was used and the cutoff distance of the van der Waals-terms were set at 12.5 Å. As a general constraint,

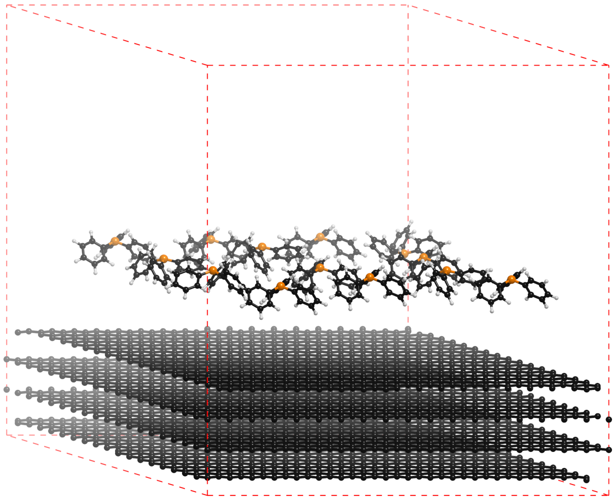

Supplementary Figure 5: Snapshot of the supercell setup for a coverage of 0.5 ML PPh_3 on graphite in material studio.

the positions of the inner two graphite layers were fixed so that real conditions were adopted and to minimise calculation time. The total simulation time was set to 30 ps using a time step Δt of 1 fs which equals a simulation of 30,000 steps. The temperatures where chosen to be at 50 K, 150 K, 298 K and 500 K, respectively.

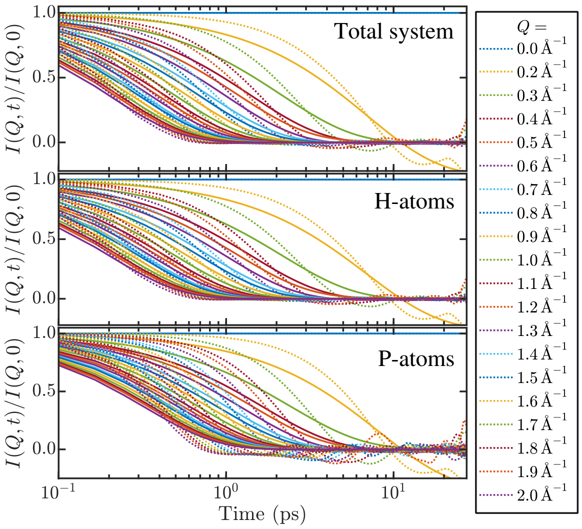

Supplementary Figure 6: Calculated ISFs from the trajectories of the total system (top), H-atoms (middle) and P-atoms (bottom), following force field MD simulations of 0.5 ML PPh_3 on graphite at 500 K. The calculated IFS are shown as dotted lines and the corresponding fits as solid lines. At small Q-values there is some deviation from the single-exponential behaviour and particular for the P-atom trajectories the ISFs tend to be "noisier" as there are only a few P-atoms in the system.

After simulating the trajectories of the particles in Materials Studio, the trajectory files were loaded into the software MDANSE. MDANSE is a Python package which was developed at the ILL and allows to calculate scattering functions from simulated trajectory files.[4] In MDANSE, the total intermediate scattering function (ISF) $I(Q,t)$ of all particles in the system was calculated numerically in order to compare the simulation to the experimental outcome. Therefore, a spherical Q-lattice in the range from 0 Å to 2 Å was created and the calculation was weighed with the incoherent scattering lengths of the particles. The instrument resolution function was also implemented as a Gaussian function using the full width at half maximum (FWHM) of the instruments where the measurements were performed. In addition we have also calculated the ISFs considering only the P- and H-atoms in the system in order to illustrate their contribution to the dynamical broadening as shown Figure 5 of the main manuscript. Therefore, the ISFs were fitted with the KWW function (equation (2) of the main manuscript), analogous to the treatment of the experimental data to extract the corresponding decay τ (see Supplementary Figure 6).

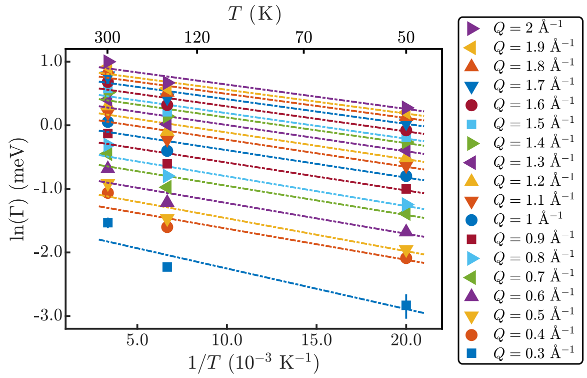

Supplementary Figure 7: Arrhenius' plot of the broadening Γ extracted from the MD simulations in the ≤ 300 K temperature region.

For the low-temperature motion from 50 K to 300 K, the activation energy for the dynamical process was also calculated from the MD simulations following Equation 3 of the main text. Supplementary Figure 7 shows an Arrhenius plot of the broadening Γ extracted from MD simulations. Upon averaging over all Q-values, we obtain a value of $E_a \approx (3.8 \pm 0.5)$ meV, thus showing a very small energy barrier for uniaxial and intramolecular rotation that dominate the dynamics in this temperature regime. The barrier is also in good agreement with the experimentally determined value of $\approx (5 \pm 2)$ meV.

Videos from the MD simulation: The force field simulations at 50 K and 500 K were saved in Materials Studio[2] as video files in order to get a real-space impression of the molecular motion. Additionally, an MD simulation for a system with a single adsorbed PPh_3-molecule was performed at 300 K using the same graphite substrate in order to visualise the intramolecular motion. In this temperature range, the molecule follows a translational motion along the graphite surface that is accompanied by a pronounced and unhindered "flapping" motion of the phenyl groups.

The other videos for the full 0.5 ML system provide further insight into the real-space motion of the system. At low temperature of 50 K, the PPh_3-molecules stay in close contact with the surface and exhibit a restricted rotational motion with very little diffusion.

In the high-temperature regime, the dynamical process changes significantly: The PPh_3-molecules do not only overcome the barrier for translational motion, in fact at 500 K most molecules even desorb at some point from the surface (some of them re-adsorb again due to periodic boundary conditions). As mentioned in the main text, we expected that it is related to an underestimation of the adsorption energy for tripod-like molecules such as PPh_3 in contrast to planar polyaromatic hydrocarbons adsorbed on graphite.

Nevertheless, in both the low and high-temperature cases, a pronounced rotational motion of the molecular phenyl groups is present.

Supplementary References

(1) Kelsall, J.; Townsend, P. S. M.; Ellis, J.; Jardine, A. P.; Avidor, N. Ultrafast Diffusion at the Onset of Growth: O/Ru(0001). *Phys. Rev. Lett.* **2021**, *126*, 155901.

(2) Dassault Systèmes BIOVIA, Materials Studio, Version 2017 R2, San Diego: Dassault Systèmes (2017).

(3) Akkermans, R. L. C.; Spenley, N. A.; Robertson, S. H. COMPASS III: automated fitting workflows and extension to ionic liquids. *Molecular Simulation* **2021**, *47*, 540–551.

(4) Goret, G.; Aoun, B.; Pellegrini, E. MDANSE: An Interactive Analysis Environment for Molecular Dynamics Simulations. *J. Chem. Inf. Model.* **2017**, *57*, 1–5.
