# Supplementary material for: Molecular motion of a nanoscopic moonlander via translations and rotations of triphenylphosphine on graphite
Source: Commun Chem. 2024 Apr 6;7:78. doi: 10.1038/s42004-024-01158-7 (PMC10998885; doi:10.1038/s42004-024-01158-7)
Supplement: Supplementary file 3 — Description of Additional Supplementary Files [file 42004_2024_1158_MOESM3_ESM.pdf]

Description of Additional Supplementary Files

File name: Supplementary Movie 1

Description: Molecular dynamics video of 0.5 monolayer at 50 K

File name: Supplementary Movie 2

Description: Molecular dynamics video of a single adsorbed molecule at 300 K

File name: Supplementary Movie 3

Description: Molecular dynamics video of 0.5 monolayer at 500 K
